# Factors Associated with Alcohol-Related Injuries for Aboriginal and Non-Aboriginal Australians: An Observational Study

**DOI:** 10.3390/ijerph17020387

**Published:** 2020-01-07

**Authors:** Mieke Snijder, Bianca Calabria, Timothy Dobbins, Anthony Shakeshaft

**Affiliations:** 1 The Matilda Centre for Research in Mental Health and Substance Use, the University of Sydney, Sydney 2006, Australia; 2 National Centre of Epidemiology and Public Health, Australian National University, Canberra 2601, Australia; bianca.calabria@anu.edu.au; 3 National Drug and Alcohol Research Centre, University of New South Wales, Sydney 2034, Australia; t.dobbins@unsw.edu.au (T.D.); a.shakeshaft@unsw.edu.au (A.S.)

**Keywords:** alcohol-related harms, aboriginal, indigenous, community, epidemiology, emergency department

## Abstract

Alcohol use and related injuries are a leading risk factor for deaths and disabilities in Australia, particularly for Aboriginal and Torres Strait Islander people. An improved understanding of individual and geographical community characteristics that are significantly associated with higher rates of alcohol-related injuries for specific populations can contribute to more effective efforts aimed at reducing alcohol-related injuries. For Aboriginal and non-Aboriginal Australians in New South Wales, this study used emergency department (ED) data to investigate rates of alcohol-related injuries, whether differences in rates vary between communities, and individual and community characteristics significantly associated with alcohol-related injuries. Differences in rates of alcohol-related injuries between Aboriginal and non-Aboriginal people varied significantly between communities. Being younger than 38 years old was significantly associated with increased risk of alcohol-related injuries, independent of Aboriginal status and gender. Increased disadvantage of the geographical community inhabited was associated with increased alcohol-related injuries for males. For Aboriginal males, living in a regional community was significantly associated with increased alcohol-related injuries, compared to living in major cities. Conversely, for non-Aboriginal people, living in regional communities was significantly associated with fewer alcohol-related injuries. It is therefore likely that an explanation for between-community differences can be found in regional communities.

## 1. Introduction

Alcohol use is a leading risk factor for deaths and disability for those younger than 50 years of age [1], particularly among disadvantaged populations. For example, in Australia, 2.3% of the burden of disease is attributed to alcohol use for the general population [1,2], but 8% for Aboriginal and Torres Strait Islander Peoples [3]. Alcohol-related intoxication can result in sustained injuries: 14% of all emergency department (ED) presentations were alcohol-related in Australia in 2013 [4]. Alcohol-related injuries have a high, preventable societal cost of between 1.3% and 3.3% of countries’ gross domestic product [5,6,7], costing the health care systems annually approximately 1.24 billion USD in Australia [8].

Individual characteristics that are significantly associated with increased alcohol-related injuries include being younger, male, unmarried and unemployed [9,10,11,12,13]. Community characteristics significantly associated with higher rates of alcohol-related injuries include socioeconomic disadvantage, increased density of alcohol outlets and remoteness of the community [9,14,15,16,17]. Previous studies have not identified whether these characteristics differ between communities in the same jurisdiction or between different groups and who may experience higher rates of alcohol-related injuries, such as Aboriginal compared to non-Aboriginal People [18]. An improved understanding of the generalisability of individual and community characteristics that are significantly associated with higher rates of alcohol-related injuries can contribute to more effective efforts aimed at reducing alcohol-related injuries as they can target the specific characteristics of different communities and defined populations.

This study had three aims. First, to estimate and specify rates of alcohol-related injuries by individual and community characteristics in New South Wales (NSW) Australia, separately for Aboriginal and non-Aboriginal Australians. Second, to investigate whether differences in rates of alcohol-related injuries between Aboriginal and non-Aboriginal Australians vary across geographical communities. Third, to identify individual and community characteristics that are significantly associated with alcohol-related injuries separately for Aboriginal and non-Aboriginal Australians.

## 2. Materials and Methods

### 2.1. Ethics

The research was approved by the NSW Aboriginal Health and Medical Research Council Ethics Committee (no. 987/13) and the NSW Population and Health Services Research Ethics Committee (no. 2014/02/516).

### 2.2. Study Design and Setting

An epidemiological study using routinely collected ED presentation data in NSW between 1 January 2012 and 31 December 2014. NSW is the most populous state of Australia (an estimated population of approximately 7.5 million), of whom 2.9% identify as Aboriginal and/or Torres Strait Islander. The majority (77%) of the NSW population resides in the major cities (defined as having a high accessibility to services and 250,000 or more inhabitants) [19].

### 2.3. Data Sources and Definitions

#### 2.3.1. Emergency Department Presentation Data

The Emergency Department Data Collection (EDDC), held by the NSW Ministry of Health, is a routinely collected dataset containing presentation data from public hospital EDs in NSW. For this study, the NSW Ministry of Health provided de-identified unit-level data for ED presentations involving persons aged 13 years and older for the period from l January 2012 to 31 December 2014 [20]. Unit-level data comprised age, gender, Aboriginal status and postcode of the patient, date and time of arrival at ED and primary diagnosis of presentation. Aboriginal status was recorded as Aboriginal, Torres Strait Islander, both Aboriginal and Torres Strait Islander, non-Aboriginal or unknown. Aboriginal status in the EDDC is based on identification in the ED presentation record. Patients with an unknown Aboriginal status were excluded from the dataset (4.9%; *n* = 811,776).

This study used a proxy measure to identify alcohol-related injuries. This proxy measure was developed and validated by Young et al. [13] internationally and in Australian EDs, which correctly identified 71% of alcohol-related presentations as such [13]. Using this proxy measure, all injury-related ED presentations occurring between 12:00 and 04:59 were coded as alcohol-related injuries. An ED presentation was classified as being injury-related if the primary diagnosis was 800–900 (ICD-9-CM), or S00–S99 and T00–T32 (ICD-10-AM). SNOMED-CT codes were mapped to ICD-10-AM codes, using a mapping table provided by the NSW Ministry of Health (included in the Supplementary Materials).

#### 2.3.2. Individual Characteristics

Individual characteristics were Aboriginal status, age and the gender of patient as identified in the ED data. Aboriginal status was recoded as Aboriginal (comprising Aboriginal, Torres Strait Islander and both) or non-Aboriginal. Gender was coded as male or female. Age was grouped into 5-year age categories, starting with 13 years old as the youngest age available in the data and allowing to split between those who are legally able to purchase and drink alcohol from those who are not (legal age is 18 years old in Australia). The resulting age categories are: 13–17, 18–22, 23–27, 28–32, 33–37, 38–42, 43–47, 48–52, 53–57, 58–62, 63–67 and 68+. Splitting up the age groups into 5-year categories provides the most detailed insight into which groups are most at risk of alcohol-related injuries.

#### 2.3.3. Community Characteristics

Community characteristics were obtained by postcode area (POA), based on the postcode of residence of the patient. Though a variety of geographical units can be applied to identify a ‘community’ in Australia, POA was preferred because postcodes were included in the ED dataset and community characteristics of interest were more readily available by POA, compared to other geographical units. Four postcodes (*n* = 86 (0.03%) alcohol-related injuries) were excluded because no data on community characteristics were available for those POAs.

*Socioeconomic status (SES)—categorical.* The SES of the geographical community was determined using the Index for Relative Disadvantage, which includes variables reflecting the relative disadvantage in an area compared to the rest of the state [21]. Each POA was categorised according to their corresponding quintile score, which were obtained from the Australian Bureau of Statistics (ABS) using 2011 POA census data: POAs in the lower quintiles were less disadvantaged and POAs in higher quintiles were more disadvantaged [22]. Quintile scores of NSW communities have been stable during the period included in this study [23].

*Remoteness—categorical.* Remoteness of the POAs was identified using the Australian Standard Geographical Classification remoteness structure, which defines remoteness based on road distance to service centres. POAs were grouped into three categories: major cities, regional and remote [24].

*Liquor licences—continuous.* Information on liquor licensing in the communities was obtained from the NSW Office of Liquor, Gambling and Racing [25]. Types of licences were split into three groups: on-venue licences (e.g., bars, clubs, and pubs), packaged licences (e.g., bottle shops) and hotel licences (which can have both an on-venue and packaged licence). For each type, the per capita rate of liquor licences per 1000 population in POAs were calculated and included in the model as continuous variables.

*Population characteristics—continuous.* For each POA, data on the proportion of young males (20–29 years old) and the proportion of Aboriginal Australians were obtained from the ABS using 2011 POA Census data, the most recent information at the time of analysis (ABS, 2011).

### 2.4. Statistical Analyses

All analyses were conducted using STATA 14 (Statacorp, College Station, TX, USA) [26]; confidence intervals were calculated at the 99% level. Age-standardised incidence rates were calculated for alcohol-related injuries, using direct standardisation to the 2001 Australian Population, as recommended by the ABS [27]. Incidence rates were calculated separately for each age group, for Aboriginal males and females and non-Aboriginal males and females. Age-standardised rates were calculated for Aboriginal and non-Aboriginal ED patients by gender and individually calculated for each SES quintile and level of remoteness.

Preliminary single-level Poisson models with alcohol-related injuries as the outcome variable identified that the effect of Aboriginal status on alcohol-related injuries was modified by gender (*p* < 0.01); therefore, all models in this study were stratified by gender. Prior to assessing community variation in the difference in rate ratios between Aboriginal and non-Aboriginal patients, the statistically significant differences in the rate ratios of alcohol-related injuries between Aboriginal and non-Aboriginal patients needed to be established. These were investigated using single-level Poisson models with alcohol-related injuries as the outcome variable and individual characteristics (age categories) and categorical community characteristics (SES and remoteness) as independent variables. To investigate the community variation in the rate ratios between Aboriginal and non-Aboriginal patients, a random intercept for POA was added to these single level Poisson models.

To identify which individual and community characteristics were significantly associated with alcohol-related injuries, rate ratios were calculated using multi-level Poisson models with alcohol-related injuries as outcome variable. These models were analysed separately for Aboriginal and non-Aboriginal patients and stratified by gender. They included individual (age) categorical community characteristics (SES and remoteness), continuous community characteristics (per capita rate liquor licences and proportion of young males and Aboriginal Australians) and a random intercept for POA.

## 3. Results

### 3.1. Rates of Alcohol-Related Injuries

A total of 125,834 alcohol-related injuries (2% of all ED presentations) were identified in NSW between l January 2012 and 31 December 2014 for patients aged 13 years and older (6611 (4.32%) Aboriginal patients and 119,223 (95.67%) non-Aboriginal patients). Table 1 shows that age-standardised rates of alcohol-related injuries per 1000 population were 32.8 (99% CI 31.1–34.5) for Aboriginal males, 24.6 (CI 23.1–26.2) for Aboriginal females, 9.6 (CI 9.5–9.6) for non-Aboriginal males and 6.4 (CI 6.3–6.5) for non-Aboriginal females. Rates of alcohol-related injuries were highest amongst 18–22-year-olds, followed by 23–27-year-olds, regardless of Aboriginal status or gender, except for Aboriginal males, where rates were comparable for 18–22-year-olds and 23–27-year-olds.

In terms of remoteness, age-standardised rates of alcohol-related injuries increased as remoteness increased for Aboriginal males and non-Aboriginal patients, but not for Aboriginal females where age-standardised rates were comparable in major city and remote areas. For Aboriginal patients, no clear pattern seemed to be observable in age-standardised rates of alcohol-related injuries by SES. For non-Aboriginal patients, age-standardised rates increased with increasing disadvantage.

### 3.2. Community Variation in Differences between Aboriginal and Non-Aboriginal Rates of Alcohol-Related Injuries

The unadjusted rates of alcohol-related injuries for Aboriginal males were estimated to be 3.96 (CI 3.80–4.13) times higher compared to non-Aboriginal males and 4.23 (CI 4.02–4.45) times higher for Aboriginal females compared to non-Aboriginal females. When the random intercept for POA was added, thus controlling for geographical community variation in the rate ratios of alcohol-related injuries, these differences dropped to 3.22 (CI 3.08–3.37) for males and 3.59 (CI 3.40–3.79) for females. This provided evidence that rate ratios of Aboriginal compared to non-Aboriginal alcohol-related injuries vary significantly between geographic communities for both males (*p* < 0.01) and females (*p* < 0.01).

### 3.3. Characteristics Associated with Alcohol-Related Injuries

Table 2 shows the characteristics that are associated with alcohol-related injuries. In terms of individual characteristics, for both Aboriginal and non-Aboriginal patients, there was an overall decreasing trend between age and alcohol-related injuries after adjusting for geographical community characteristics. In terms of geographical community characteristics, for Aboriginal and non-Aboriginal males, living in relatively disadvantaged communities (Quintiles 2–5) was significantly associated with increased alcohol-related injuries compared to living in less disadvantaged communities (Quintile 1). For non-Aboriginal people, living in regional communities was significantly associated with fewer alcohol-related injuries compared to living in major cities. For Aboriginal males, living in regional communities was significantly associated with increased alcohol-related injuries compared to living in major cities.

A higher per capita rate of hotel licenses was associated with fewer alcohol-related injuries for non-Aboriginal people and Aboriginal males. A higher per capita rate of on-venue licenses was associated with increased alcohol-related injuries for non-Aboriginal females. A higher percentage of young males in a community was associated with increased alcohol-related injuries, regardless of gender and Aboriginal status.

## 4. Discussion

This study estimated rates of alcohol-related injuries and found similar trends for Aboriginal and non-Aboriginal Australians across individual and community characteristics. While Aboriginal Australians experienced significantly higher rates of alcohol-related injuries than non-Aboriginal people, the extent to which these rates differed varied between geographical communities. Young people (aged 18–27 years old) were most likely to experience alcohol-related injuries. For non-Aboriginal Australians, regional communities were associated with lower rates of alcohol-related injuries compared to major cities, but rates of alcohol-related injuries increased for Aboriginal males in regional communities. Increased disadvantage was associated with increased alcohol-related injuries for Aboriginal and non-Aboriginal males. Communities with a higher percentage of young males had higher rates of alcohol-related injuries for Aboriginal and non-Aboriginal people.

The extent to which rates of alcohol-related injuries differed between Aboriginal and non-Aboriginal people varied between communities. This was true for males and females, all age groups and all communities, regardless of remoteness and SES. The analyses presented in Table 2 identified few community-level characteristics with significantly and practically meaningful differences between Aboriginal and non-Aboriginal Australians that might explain why the rate ratio of alcohol-related injuries for Aboriginal, compared to non-Aboriginal, differed significantly between communities. The only differences (where the rate ratios for Aboriginal and non-Aboriginal people were consistently in the opposite directions) were for geographical location of the community, specifically: for Aboriginal males, regional communities were consistently associated with more alcohol-related injuries (relative to Aboriginal males in major cities) and for non-Aboriginal people, regional communities were consistently associated with fewer alcohol-related injuries (relative to non-Aboriginal people in major cities). This indicates that, to address the disparity in alcohol-related injuries between Aboriginal and non-Aboriginal people, focusing on regional communities might have the largest impact as that is where the differences between Aboriginal and non-Aboriginal people seem greatest.

It is likely that other factors not measured in this study contribute to higher rates of alcohol-related injuries experienced by Aboriginal people. Previous studies into risk factors for non-alcohol-specific injuries have identified a range of risk factors that are unique to Indigenous Peoples both in Australia and internationally. These risk factors include historical factors (i.e., the impact of colonisation and dispossession), disproportionately high experience of social stressors at a younger age (e.g., death of a loved one, family break ups, financial stress, racism) [28] and community tensions that are largely a consequence of forced relocation of Indigenous families coinciding with European colonisation [29]. As some of these factors are also known to exacerbate alcohol consumption [30], it is likely that these factors contribute to alcohol-related injuries as well but were not measured in this study. It has been acknowledged that policies and programs for Aboriginal Australians should focus on these contextual factors of historical trauma, colonisation, racism and present-day social conditions [31,32].

The observation that alcohol-related injuries were significantly lower in regional areas for non-Aboriginal Australians is surprising considering increased remoteness has been associated with increased high risk drinking and other alcohol-related harms, such as assaults [9,17,18,33]. It is possible that this effect is driven by more hotel licences per capita in more regional communities, as a higher per capita rate of hotel licences was significantly associated with fewer alcohol-related injuries for non-Aboriginal people, and regional communities have significantly more hotel licenses per capita compared to major cities. Another potential explanation is that the EDDC data used in this study do not comprehensively cover all NSW EDs [34]. With mainly smaller, regional and remote EDs not supplying data to the EDDC. There are therefore fewer datapoints available for regional and remote EDs potentially resulting in less data available in the dataset used for the current study.

In line with previous research, this study clearly highlights the need for alcohol-related injury prevention efforts to continue to target young people [9,11,33,35]. Previous research has identified that increasing the minimum age of drinking from 18 (which it currently is in Australia) to 21, for example, has the potential to significantly reduce alcohol-related harms for young people aged 18–21, but also for those 21–25 [36,37]. Evidence suggests reductions in supply and availability of alcohol [38] as well as changing norms around adolescent drinking [39] have the potential to contribute to the reduction in rates of alcohol-related injuries experienced by young people. In addition, recent research has sought to improve methods for co-designing community-based programs aimed at reducing alcohol-related injuries and harms, including for young people [40], which have the potential to reduce alcohol-related harms.

### Methodological Limitations

While the use of administrative data to monitor and assess alcohol-related outcomes is beneficial [41,42], there are a number of methodological limitations relating to using these kinds of data that are worth noting and potentially influence the confidence in the findings of this study.

Firstly, the EDDC data do not include all EDs in NSW. Between 2011–2012 and 2014–2015, the EDDC covered between 97% and 99% of metropolitan EDs and 81–94% of rural and regional EDs. It does not include private hospitals, and it excludes some smaller and remote EDs [34,43,44]. Missing these hospitals in the dataset may have influenced the effect estimates.

Second, the use of a proxy measure might not be the ideal way of identifying alcohol-related injuries in EDs, however it is currently identified the most reliable method [35]. While there may be options for ED personnel to record the involvement of alcohol as external cause to the injury, this is not always documented due to a lack of time to conduct appropriate testing (e.g., blood or breath tests), more pressing pathology, or ED staff not being aware of this option or being too busy to report external causes [35,45]. Even where alcohol may be recorded as an external cause in the source system, it is not available in the EDDC. Where the involvement of alcohol cannot be directly extracted from the data, the use of a temporal proxy measure is recommended [13,46,47].

Further contributing to issues with the proxy measure is the less than optimal reliability of the primary diagnosis used to identify injury-related presentations. The accuracy of the primary diagnosis is at least 85% for ED patients subsequently admitted to the hospital (27% of all patients) [35]. The reliability is diminished for non-admitted patients; however, validity of primary diagnoses for injury presentations is higher than that of other medical conditions because of the more obvious pathology [35]. This is due to diagnoses being entered by ED personnel who are not necessarily trained in clinical coding and the use of three different computer programs in EDs across NSW with different methods of entering diagnostic codes [35].

The proxy measure used in this study has not been explicitly validated for Aboriginal Australians. Proxy measures have been widely used in previous research into community-level alcohol-related ED presentations for the general population in Australia, which includes Aboriginal Australians [13,48,49], but this is the first study to use the proxy measures to investigate alcohol-related injuries specifically for Aboriginal Australians with ED data. Further work on validating proxy measures for Aboriginal Australians is required [50]. Furthermore, Aboriginal status identification is not optimal. Efforts are undertaken to increase the reliability of Aboriginal status information in administrative health datasets, and through data linkage, identification has improved from 44.1% to 74.5% between 2005 and 2014.

Finally, this paper likely does not paint a complete picture of alcohol-related injuries due to its use of only one dataset in one Australian state. Alcohol-related injuries are also reported to general practice, ambulance services and other primary health care settings. Triangulating data from these data sources can further improve our understanding as to what contributes to increased alcohol-related injuries. Further similar studies in other parts of Australia might provide a completer picture of what contributes to alcohol-related injuries amongst Aboriginal Australians.

## 5. Conclusions

This study used administrative data to assess alcohol-related injury presentations to EDs for Aboriginal and non-Aboriginal people in NSW. This study showed that rates of alcohol-related injuries are higher among Aboriginal Australians than non-Aboriginal Australians, but that these differences vary between community. A potential explanation for this can be found in regional communities, where rates of alcohol-related injuries are increased for Aboriginal males but decreased for non-Aboriginal people. Therefore, initiatives are required to target reductions in alcohol misuse and injuries for non-Aboriginal and Aboriginal males. The target areas for these should be driven by data; given findings that there is community variation, this study indicates a need in both urban and regional settings. Prevention efforts targeting young people are required.

## Figures and Tables

**Table 1:** Total number and age-standardised rates of alcohol-related injuries in New South Wales (NSW) between 1 January 2012 and 31 December 2014, by Aboriginal status and gender.

Alcohol-Related Injuries	Aboriginal						Non-Aboriginal					
	Male			Female			Male			Female		
	N	ASR ^a^	(99% CI)	N	ASR ^a^	(99% CI)	N	ASR ^a^	(99% CI)	N	ASR ^a^	(99% CI)
**Total Alcohol-Related Injuries**	3940	32.8	(31.1–34.5)	2671	24.6	(23.1–26.2)	70,119	9.6	(9.5–9.6)	49,104	6.4	(6.3–6.5)
**Individual Characteristics**												
**Age Group**												
**13–17**	607	25.7	(23.1–28.5)	464	21.6	(19.1–24.3)	6018	10.0	(9.7–10.3)	4660	8.2	(7.9–8.5)
**18–22**	962	49.3	(45.3–53.6)	571	34.7	(31.1–38.6)	13,688	22.5	(22.0–23.0)	7393	12.8	(12.4–13.1)
**23–27**	669	49.7	(44.9–54.9)	359	31.3	(27.2–35.8)	10,463	17.1	(16.7–17.5)	5449	8.8	(8.5–9.1)
**28–32**	432	42.4	(37.3–47.9)	275	28.9	(24.6–33.7)	7989	12.6	(12.2–13.0)	4308	6.6	(6.3–6.9)
**33–37**	330	40.1	(34.6–46.1)	223	28.1	(23.5–33.3)	6112	9.8	(9.5–10.2)	3375	5.3	(5.1–5.6)
**38–42**	299	32.4	(27.8–37.5)	241	23.8	(20.0–28.1)	5434	8.2	(7.9–8.5)	3341	4.8	(4.6–5.0)
**43–47**	246	30.9	(26.0–36.3)	208	24.6	(20.5–29.4)	4288	6.9	(6.7–7.2)	3098	4.9	(4.7–5.1
**48–52**	174	30.3	(24.7–36.7)	130	21.8	(17.2–27.3)	3807	6.0	(5.7–6.2)	3035	4.6	(4.4–4.8)
**53–57**	114	31.4	(24.3–39.8)	83	22.8	(16.8–30.0)	2943	5.1	(4.9–5.3)	2612	4.4	(4.2–4.6)
**58–62**	54	25.3	(17.3–35.6)	54	21.6	(14.8–30.4)	2253	4.4	(4.2–4.6)	2080	3.9	(3.7–4.1)
**63–67**	22	21.4	(11.5–36.2)	22	20.5	(11.0–34.7)	1952	4.3	(4.1–4.6)	1737	3.8	(3.6–4.0)
**68+**	31	14.4	(8.6–22.6)	41	16.4	(10.5–24.2)	5172	5.2	(5.0–5.4)	8016	6.4	(6.2–6.6)
**Community Characteristics**												
**Remoteness**												
**Major City**	1198	32.3	(28.3–36.3)	823	24.8	(21.5–28.2)	45,587	8.6	(8.5–8.7)	32,675	5.9	(5.8–6.0)
**Regional**	2482	33.6	(31.5–35.7)	1609	23.9	(22.0–25.7)	23,700	12.1	(11.8–12.3)	15,952	7.6	(7.4–7.8)
**Remote**	260	35.3	(28.2–42.4)	239	24.7	(28.1–41.2)	832	15.2	(13.8–16.5)	477	10.0	(8.7–11.2)
**SES Quintile**												
**1 (Least Disadvantaged)**	2310	42.2	(17.2–67.4)	61	25.1	(15.7–34.5)	13,325	7.5	(7.3–7.7)	10,393	5.4	(5.3–5.6)
**2**	317	30.3	(21.3–39.4)	239	29.3	(22.6–36.1)	13,812	8.6	(8.5–8.8)	9819	6.0	(5.8–6.1)
**3**	1206	31.9	(29.0–34.7)	748	23.1	(20.3–26.0)	17,424	10.3	(10.1–10.5)	12,095	6.9	(6.7–7.0)
**4**	1112	34.1	(30.6–37.6)	707	21.9	(19.3–24.5)	12,598	11.3	(11.1–11.6)	8462	7.2	(7.0–7.4)
**5 (Most Disadvantaged**	1214	33.8	(30.6–37.0)	916	27.8	(24.8–30.7)	12,960	11.4	(11.2–11.7)	8335	7.0	(6.8–7.2)

N = total number of alcohol-related injuries; ^a^ ASR = age-standardised rates, per 1000 population; CI = confidence interval; SES = socioeconomic status.

**Table 2:** Adjusted rate ratios for multi-level Poisson regression model of alcohol-related injuries among Aboriginal and non-Aboriginal persons in NSW.

Gender	Aboriginal (N = 13,987)				Non-Aboriginal (N = 283,469)			
	Males		Females		Males		Females	
	RR ^a^	99% CI	RR ^a^	99% CI	RR ^a^	99% CI	RR ^a^	99% CI
**Individual Characteristics**								
***Age Patient***								
13–17	1.60 *	(1.32–1.93)	1.42 *	(1.14–1.76)	1.10 *	(1.05–1.15)	1.53 *	(1.31–1.48)
18–22	2.28 *	(1.91–2.73)	1.87 *	(1.52–2.30)	2.47 *	(2.36–2.57)	2.18 *	(2.06–2.31)
23–27	1.71 *	(1.42–2.06)	1.31 *	(1.05–1.64)	1.92 *	(1.83–2.00)	1.67 *	(1.58–1.76)
28–32	1.28 *	(1.04–1.56)	1.06	(0.84–1.34)	1.47 *	(1.41–1.54)	1.31 *	(1.23–1.40)
33–37	1.03	(0.83–1.28)	0.99	(0.78–1.28)	1.12 *	(1.07–1.18)	1.03	(0.97–1.10)
38–42	1.00		1.00		1.00		1.00	
43–47	0.80	(0.63–1.00)	0.92	(0.72–1.19)	0.79 *	(0.75–0.84)	0.94	(0.88–1.01)
48–52	0.71 *	(0.55–0.92)	0.71 *	(0.53–0.96)	0.71 *	(0.67–0.75)	0.92 *	(0.86–0.98)
53–57	0.58 *	(0.43–0.78)	0.58 *	(0.41–0.81)	0.54 *	(0.51–0.58)	0.80 *	(0.75–0.86)
58–62	0.44 *	(0.30–0.65)	0.48 *	(0.32–0.72)	0.43 *	(0.40–0.45)	0.63 *	(0.59–0.68)
63–67	0.36 *	(0.15–0.46)	0.33 *	(0.19–0.60)	0.36 *	(0.34–0.39)	0.54 *	(0.50–0.59)
68+	0.32 *	(0.20–0.54)	0.48 *	(0.31–0.77)	0.95	(0.90–1.00)	2.35 *	(2.22–2.49)
**Community Characteristics**								
***SES***								
1 (Least Disadvantaged) (ref)	1.00		1.00		1.00		1.00	
2	1.20	(0.69–2.08)	1.26	(0.73–2.17)	1.17	(0.83–1.69)	1.13	(0.82–1.56)
3	1.71 *	(1.02–2.86)	1.54	(0.91–2.59)	1.45 *	(1.03–2.05)	1.36	(0.99–1.87)
4	1.75 *	(1.03–2.97)	1.48	(0.87–2.53)	1.47 *	(1.02–2.13)	1.30	(0.92–1.84)
5 (Most Disadvantaged)	1.65	(0.96–2.84)	1.54	(0.90–2.64)	1.56 *	(1.07–2.27)	1.30	(0.92–1.85)
***Remoteness***								
Major City (ref)	1.00		1.00		1.00		1.00	
Regional	1.46 *	(1.05–2.03)	1.55	(1.13–2.11)	0.64 *	(0.47–0.87)	0.65 *	(0.49–0.87)
Remote	1.16	(0.57–2.38)	1.92	(0.98–3.79)	0.54	(0.28–1.07)	0.65	(0.32–1.29)
***Liquor Licenses***								
Hotels	0.65 *	(0.47–0.91)	0.77	(0.57–1.02)	0.49 *	(41.5–58.2)	0.50 *	(0.42–0.59)
On-Venue	1.02	(0.91–1.14)	0.99	(0.89–1.11)	1.07	(99.7–1.14)	1.08 *	(1.02–1.16)
Packaged	1.04	(0.88–1.21)	0.99	(0.85–1.14)	0.93	(0.81–1.06)	0.91	(0.80–1.04)
***Population Characteristics ^#^***								
Young Males	1.07 *	(1.00–1.14)	1.07 *	(1.00–1.14)	1.11 *	(1.06–1.17)	1.08 *	(1.03–1.14)
Aboriginal	1.02	(0.99–1.04)	1.00	(0.98–2.64)	0.98	(0.96–1.00)	0.98	(0.96–1.01)

^a^ Adjusted incidence rate ratios; ^#^ Percentage of young males and Aboriginal Australians per POA; * *p* < 0.01.

## Supplementary Materials

The following are available online at https://www.mdpi.com/1660-4601/17/2/387/s1, SNOMED CT to ICD10 mapping table.
